# A Miniaturized Method for Evaluating the Dynamic Gas-Phase Adsorption and Degradation of Sarin on Porous Adsorbents at Different Humidity Levels

**DOI:** 10.1021/acsomega.4c02306

**Published:** 2024-06-21

**Authors:** Lillemor Örebrand, Linnea Ahlinder, Marianne Thunéll, Robin Afshin Sander, Andreas Larsson, Andreas Fredman, Håkan Wingfors

**Affiliations:** CBRN Defence and Security, Swedish Defence Research Agency, 901 82 Umeå, Sweden

## Abstract

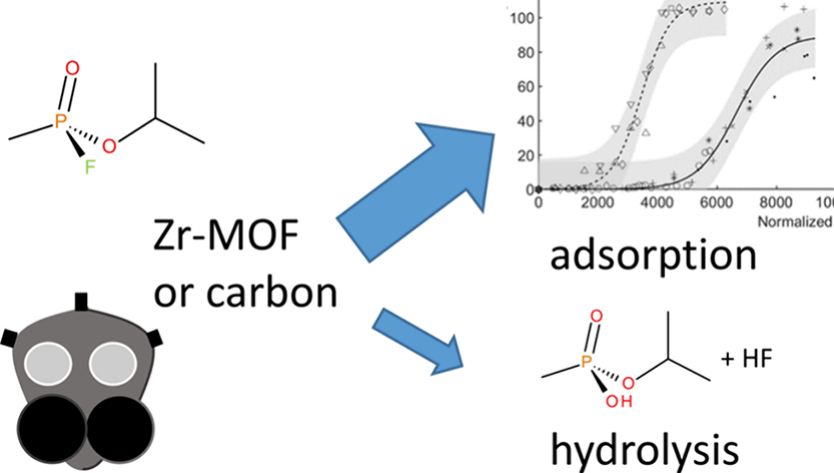

Metal organic frameworks based on zirconium nodes (Zr-MOFs) have impressive adsorption capacities, and many can rapidly hydrolyze toxic organophosphorus nerve agents. They could thus potentially replace commonly used adsorbents in respiratory filters. However, current test methodologies are poorly adapted to screen the large number of available MOFs, and data for nerve agent adsorption by MOFs are scarce. This paper presents a miniaturized method for assessing the capacity of Zr-MOFs for dynamic gas phase adsorption and degradation of sarin (GB) into the primary hydrolysis product isopropyl methyl phosphonic acid (IMPA). The method was validated by comparing the dynamic adsorption capacities of activated carbon (AC) and NU-1000 for GB under dry and humid conditions. Under dry conditions, unimpregnated AC had a greater capacity for GB uptake (0.68 ± 0.06 g/g) than pelletized NU-1000 (0.36 ± 0.03 g/g). At 55% relative humidity (RH), the capacity of AC was largely unchanged (0.72 ± 0.10 g/g) but that of NU-1000 increased slightly, to 0.46 ± 0.10 g/g. However, NU-1000 exhibited poor water retention at 55% RH. For both adsorbents, the degree of hydrolysis of GB into IMPA was significantly greater at 55% RH than under dry conditions, but the overall degree of hydrolysis was limited in both cases. Further tests at higher relative humidities are needed to fully evaluate the ability of NU-1000 to degrade GB after adsorption from the gas phase. The proposed experimental setup uses very small amounts of both adsorbent material (20 mg) and toxic agent, making it ideal for assessing new MOFs. However, future methodological challenges are reliable generation of sarin at higher RH and exploring sensitive methods to monitor degradation products from nerve agents in real-time.

## Introduction

The organophosphorus (OP) nerve agents sarin (GB), soman (GD), cyclosarin (GF) and O-ethyl S-(2-diisopropylaminoethyl)phosphonothiolate (VX) are among the most toxic chemical warfare agents (CWA) to humans because of their ability to block the function of the enzyme acetylcholinesterase in synapses. In 1997 the Chemical Weapons Convention (CWC) came into force, aiming to eliminate all chemical weapons by prohibiting their use, development, production, and stockpiling. Despite a worldwide commitment to the convention, recent incidents involving the use of OP nerve agents show that they remain an ongoing threat in war, terror, and crime.^1,2^ The main routes of exposure to nerve agents are dermal uptake and inhalation of vapor or aerosolised droplets.

Since World War I, activated carbon (AC) has been the primary choice for gas filtration media in respiratory protection, and later also as an adsorptive layer in protective clothing, due to its low cost, accessibility in particle sizes providing favorable flow resistance, and universal ability to adsorb OP-nerve agents and many other CWAs. Removal of volatile nerve agents from gas streams by AC is assumed to occur primarily by physisorption in the micro- and meso- pores of its semicrystalline structure.^3^ Aerosolised droplets are likely to initially be captured in the particle filter of combined filter cartridges and to then evaporate slowly, leading to capture of the CWA in the activated carbon. Contaminated AC in respirators and clothing layers may present a risk of secondary contamination via evaporation from canisters or direct skin contact with clothing, at least on relatively short time scales. Previous studies on OP nerve agents adsorbed onto AC have shown that degradation into the less toxic alkyl phosphonic acids is slow (*t*_1/2_ > 5 days)^4,5^ unless the material is treated and regenerated with heat, water, and/or reactive chemicals to reduce the half-life of the CWA to hours.^6,7^ Conventional decontamination methods for nerve agents based on incineration or treatment with excess base in water are effective^8^ but less suitable for material recovery. Adsorbents capable of promoting a “self-detoxifying” degradation process for adsorbed CWAs would thus be highly desirable for future protective equipment.

Porous crystalline metal–organic frameworks (MOFs) and other porous adsorbents have attracted great interest over the past decade. These materials have demonstrated both competitive adsorption capacity for nerve agents and the ability to catalyze their hydrolytic degradation.^9−14^ Recent studies on MOF powders based on zirconium nodes (Zr_6_(μ_3_-O)_4_(μ_3_–OH)_4_) and various organic linkers have shed light on the factors controlling hydrolysis, allowing OP nerve agents and simulants to be degraded at increasingly impressive rates (*t*_1/2_ < 1 min).^15,16^ The rate of catalytic hydrolysis can be enhanced by introducing missing linkers or defect nodes to increase accessibility to Lewis acidic metal sites, enlarging the pore apertures (e.g., by using longer linkers), and/or introducing basic moieties (e.g., NH_2_-groups) into the framework. In accordance with these findings, a recently proposed mechanism suggests that complete catalytic conversion involves several critical and consecutive steps.^15^ After entering a pore within the adsorbent, the agent must coordinate to a Lewis acidic site at a Zr-node in a manner that enables nucleophilic attack on the phosphorus center by an adjacent hydroxyl group.^17^ This weakens the P–F bond, allowing the elimination of HF. Finally, the alkylphosphonic acid must be dislocated from the site via a base-catalyzed reaction followed by restoration of the catalytic site to permit coordination of a new substrate molecule. Regardless of the exact mechanism in each step, data from several studies indicate that a catalytic reaction occurs in buffer solutions when both water and an exogenous base are present.^11,12,16,18−21^ The catalytic hydrolysis of OP nerve agents outside solution is less well supported, and the kinetics of this process are reported to be much slower than in solution.^22,23^ Nevertheless, recent advances and modifications have yielded valuable insights that suggest a practical process may be possible. For instance, it was shown that the solid state reaction can be enhanced by selecting suitable Zr-MOFs,^24^ prewetting the adsorbent/catalyst,^25^ introducing structural features that enhance water adsorption within the MOF,^26^ and fortifying the structure with suitable bases.^22,23,26,27^ Furthermore, several techniques for integrating MOFs onto fibers or polymers suitable for use in air filtration or textile membranes have been explored.^28−31^

Since AC-based filters and adsorptive layers for clothing are designed to remove toxic agents, any self-detoxifying process involving new protective materials must eventually be tested under realistic circumstances. For AC, this is often achieved by employing dynamic adsorption test systems that use less toxic simulants.^32^ However, to our knowledge, there are no published data on the inherent capability of AC to degrade nerve agents on short time scales. Presumably, this is at least partly because the implementation of dynamic test methodologies that involve applying highly toxic and reactive agents to powdered materials presents several significant challenges.

This work addresses these issues by introducing a reproducible and reliable method for assessing the adsorptive and detoxifying capacity of Zr-MOFs for nerve agents in the solid state. The method uses very small amounts of both OP-nerve agents and Zr-MOFs to reduce test times and toxic hazards, and thus facilitates fast screening of new materials. The method’s development included measurements of the capacity of the Zr-MOF NU-1000 and nonimpregnated AC to adsorb and degrade GB under both dry and humid conditions. NU-1000 is a promising Zr-MOF with large pores, whose ability to adsorb and degrade OP-agents has been studied previously.^12,24,33,34^

## Materials and Methods

CWAs such as GB are extremely toxic substances that are only allowed to be produced, stored, and handled in special laboratories, i.e. Schedule 1 facilities approved by Organisation for the Prohibition of Chemical Weapons (OPCW). Due to their toxicity, these substances can only be handled by trained personnel working in a glovebox or a very efficient fume hood and using proper personal protective equipment. GB was produced in house with >98% purity (confirmed by H^1^ NMR). Further details concerning the chemicals used in this work are presented in the Supporting Information (SI).

NU-1000 was synthesized in house following the protocol of Wang et.al,^35^ yielding a yellow powder that was carefully pelletized at low pressure, grinded, and sieved to obtain a 125–250 μm (120–60 mesh) fraction. The resulting material was characterized using powder X-ray diffraction (PXRD), attenuated total reflectance Fourier-transform infrared spectroscopy (ATR FTIR), scanning electron microscopy (SEM), and N_2_–Brunauer–Emmett–Teller (BET) analysis. In all cases, the obtained data were conforming to with previous reports. Unimpregnated granulated AC derived from coconut shells (Addsorb GA, Jacobi) was also ground and sieved to the same size fraction and used for comparative purposes. Steel tubes with an inner diameter (i.d.) of 4 mm were loaded with 20 mg of adsorbent. Consistent adsorbent packing was ensured by measuring the pressure drop over these tubes at several flow rates. Detailed descriptions of the material preparation procedures and instruments used for characterization, verification, and analysis are presented in the SI.

### Experimental Setup

**Figure 1:**
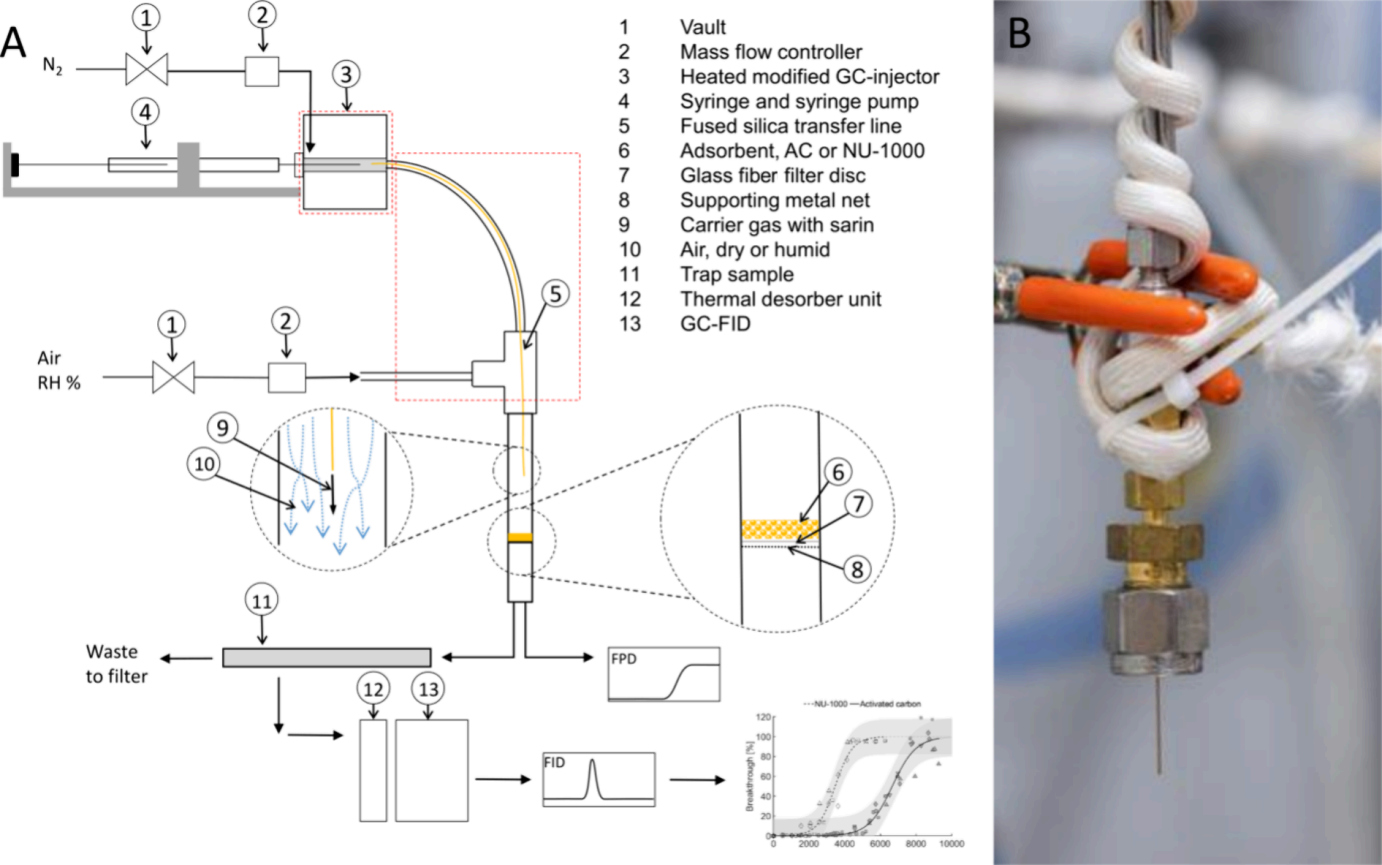
(A) Schematic depiction of the test setup with the adsorbent being tested packed in a 4 mm i.d. steel tube with a supporting metal mesh and a glass fiber filter disc. GB was vaporized in a modified heated GC-injector at 180 °C, and the vapor was transferred to the steel sample tube using a gas flow of 8 mL/min comprising an N_2_ carrier gas flow of 4 mL/min mixed with a 4 mL/min flow of dry or humid air. Downstream, an FPD was used to monitor breakthrough, and trap samples were collected on Tenax repeatedly for breakthrough analysis using a thermal desorber unit coupled to a gas chromatograph with a flame ionization detector. The dashed red rectangles indicate areas heated to different temperatures. (B) Photo of the 1/16′′ T-piece with coiled heating tape that was used to keep the temperature at 60 °C. A transfer line going through the T-piece was introduced inside the steel tube from the top. Dry or humid air was introduced via the side of the T-piece.

A schematic depiction of the experimental setup is shown in Figure 1 A. GB gas was generated from liquid in a modified heated GC-injector (at 180 °C) connected to a syringe pump operating at a pump rate of 0.092 μL/min using a 50 μL gastight Hamilton syringe (#1705N) and a carrier gas flow (N_2_) of 4 mL/min. A deactivated fused silica column (0.25 mm i.d.) was used as transfer line inside a supporting metal tube (1/16′′) for the carrier gas to convey GB into the prepared sample tube containing the adsorbent. The transfer line was passed through a T-piece (1/16′′ Swagelok) where a 4 mL/min flow of dry or humid air was allowed to mix with the agent flow. The total gas flow was thus 8 mL/min, yielding a theoretical GB concentration of 12000 mg/m^3^ (i.e., around 2000 ppm, P/P_sat_ = 0.57 at 21 °C). Flows were checked daily and a flame photometric detector (FPD, AP2C, Proengin, France) was used to check for leaks. The supporting metal tube, the tube with dry or humid air, and the T-piece were all kept at 60 °C using heating tape coiled around the tubes and T-piece (see Figure 1B). The sample tube containing the adsorbent was kept at ambient temperature (21 °C). The tube was connected to the T-piece with the transfer line reaching 22 mm into the sample tube, giving a distance of 50 mm between the tip and the packed adsorbent. The packed sorbent mass was 20 mg, giving a bed height of around 2 mm. Downstream of the sorbent, a FPD was used to visually monitor breakthrough. Trap samples were consecutively collected on Tenax TA for quantitative analysis of GB using thermal desorption gas chromatography flame ionization detection (TD-GC/FID, TD-100 xr Markes International, UK, and trace GC 1300, Thermo Scientific, USA). During the breakthrough tests, trap samples were collected by connecting the trap sample directly to the adsorbent tube using a 1/4” union from Swagelok. All trap samples collected under humid conditions were purged with dry air for 2 min at 50 mL/min to remove excess water and reduce the risk of GB hydrolysis between sampling and analysis. Data for the trap samples were used to plot breakthrough curves.

When collecting trap samples to verify challenge concentrations, a 39 mm long 4 mm i.d. empty steel tube was placed between the T-piece and the sample tube to ensure that the distance between the mixing point and the sorbent in the sample tube was similar to that in the other analyses. Tests were performed to investigate the stability of the influent concentration by taking repeated samples over 2 h. For details of the sampling, instruments, and instrument methods see the SI.

Under humid conditions, the sample tubes were prehumidified on-tube with a total volume of 1 L humid air (50–60% relative humidity (RH) at 40 °C, 50 mL/min). The humid air flow was created using a Controlled Evaporator Mixer (CEM) in a setup described in the SI. The same CEM setup was used during the breakthrough tests, giving a humid air flow of 4 mL/min into the T-piece. The theoretical water content delivered to the adsorbent tube was 83 μg water/min, corresponding to 55% RH at room temperature. The effect of humid airflow on prehumidified tubes over time was evaluated by weighing the adsorbent phase loadings every 20 min during 280 min runs.

### Hydrolysis Measurements

After each breakthrough test, each sample of the saturated adsorbent materials was transferred into glass vials containing 2 mL of anhydrous acetonitrile and ultrasonicated for 20 min. All samples were stored in a freezer (−20 °C) until analysis. Upon analysis, a fraction of the sample (from the clear phase) was diluted for further analysis. For each sample, two aliquots were prepared, one for GB analysis and one for analysis of the primary GB degradation product isopropyl methylphosphonic acid (IMPA). Both were spiked with an internal standard (IS), then the GB/IS quota was determined by GC-MS and the IMPA/IS quota by LC-MS. Further details concerning instruments, parameters, calibration, and methods are available in the SI.

### Breakthrough Curves

Breakthrough curves were fitted to the measured data after normalization by dividing all concentrations by the current challenge concentration and sorbent mass. The curves were assumed to be S-shaped ranging from 0 to 100%, so nonlinear least-squares curve fitting was used to solve for the parameters of the logistic function (L: supremum of function values, k: curve steepness, x0: the function’s midpoint). To facilitate comparisons, each breakthrough curve was normalized to reach 100% breakthrough (L = 100%). All calculations were performed in MatLab R2023a (MathWorks, US). The GB masses at 50% breakthrough was estimated by multiplying the syringe pump rate with GB density (1.09 g/cm^3^_)_ and time points were obtained from the fitted functions. Adsorption capacities where thereafter obtained by dividing the GB masses with sorbent mass. The curves were also fitted toward the semiempirical Wheeler-Jonas equation^36^ to calculate the dynamic adsorption capacity *W*_*e*_ (g/g adsorbent) for comparison purposes. Data for these calculations are found in SI.

## Results and Discussion

To obtain reproducible gas phase adsorption data for the miniaturized system and determine the degradation rate, a number of observations were made and investigated. The following sections present and discuss the experimental results and highlight key findings.

### Stability of Challenge Concentration

The challenge concentration after the T-piece (Figure 1) was measured every 15 min over 2 h under both dry and humid conditions, revealing that the concentration remained stable with minimal variation throughout. The challenge concentration was also measured before and directly after terminating each breakthrough test using Tenax tubes. The results of these measurements were equally reproducible (average coefficient of variation (CV)=3.4%, n = 30). However, the reproducibility of the challenge concentration over the full experimental period was lower (CV 18%). The average concentrations were 9700 ± 2000 mg/m^3^ (P/P_sat_ = 0.48) and 9400 ± 1200 mg/m^3^ (P/P_sat_ = 0.47) under dry and humid conditions, respectively. The measured challenge concentration of GB was lower than the theoretical value of 12000 mg/m^3^ under both dry and humid conditions (by 81% and 78%, respectively).

It was necessary to strike a delicate balance between the syringe feed rate, injector temperature, and carrier gas flow rate (4 mL/min) to achieve quantitative transfer of the agent to the adsorbent in the test tube while simultaneously accommodating a 4 mL/min downstream auxiliary flow of humid air. Unbalanced settings of any of these parameters could cause droplet formation, condensation, and binding to active sites within the injector, and might also present a risk of unwanted hydrolysis under humid conditions. For instance, raising the injector temperature above 200 °C delayed the agent feed between sample runs, probably due to untimely evaporation within the needle. However, using lower temperatures in the injector reduced the measured concentrations early in the run. To minimize problems related to binding at active sites, a deactivated liner and an uncoated deactivated fused silica column were used to create an inert path for the agent. Tests performed without the column resulted in near-complete loss of GB under humid conditions. Such deactivated columns are often used to facilitate sample introduction of reactive compounds in gas chromatography.^37^ It was also found that concentrations were more stable when the distance between the liner tip and the sorbent was 50 mm rather than 65 mm.

### Breakthrough Experiments with Activated Carbon and NU-1000 under Dry Conditions

**Figure 2:**
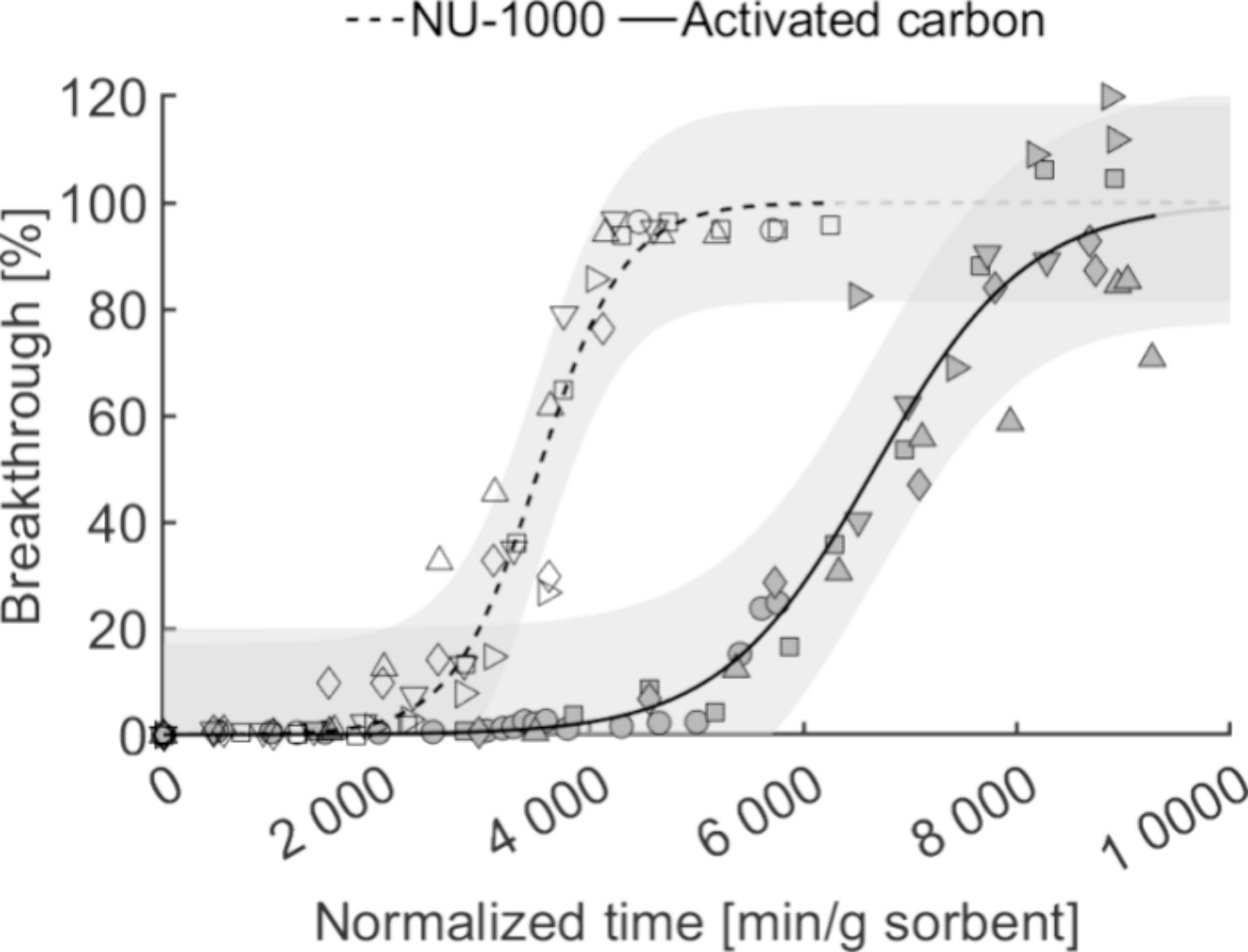
GB breakthrough curves for activated carbon (6 data series, filled symbols) and NU-1000 (6 data series, unfilled symbols) under dry conditions. The gray regions correspond to the 95% confidence intervals for each adsorbent.

All GB breakthrough measurements for activated carbon and NU-1000 were obtained using 20 mg of the relevant adsorbent. Table 1 shows the adsorbent mass and pressure drop (at 8 mL/min) for packed columns, the GB load at 50% breakthrough (calculated using the breakthrough curves shown in Figure 2), and the gravimetrically determined GB load after termination of each test.

**Table 1:** Dynamic GB Adsorption Capacities for Activated Carbon (AC) and NU-1000 under Dry Conditions

Sorbent	Mass (mg)	Pressure drop at 8 mL/min (Pa)	Adsorption capacity at 50% breakthrough^a^ (g/g)	GB load at terminated test^b^ (g/g)	Fitted function	Wheeler–Jonas *W*_*e*_^c^ (g/g)
AC (*n* = 6)	20.72 ± 1.25	56 ± 30	0.68 ± 0.06	0.56 ± 0.03	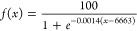	0.47 ± 0.06
NU-1000 (*n* = 5)	19.45 ± 1.29	62 ± 31	0.36 ± 0.03	0.28 ± 0.01	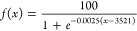	0.24 ± 0.03

a GB load calculated at 50% breakthrough.

b Gravimetrically determined.

c Calculated dynamic adsorption capacity.

Figure 2 shows the results of breakthrough measurements under dry conditions for activated carbon and NU-1000 with fitted symmetrical S-shaped breakthrough curves. GB retention by AC is assumed to occur primarily via nonspecific adsorption, the extent of which correlates with the adsorbent’s surface area, pore volume, and pore-filling mechanisms.^3,38^ The AC used in this study had a BET surface area of 1495 m^2^/g according to the manufacturer. Its adsorption capacity was determined to be 0.68 g GB/g based on the breakthrough curve value at 50% breakthrough and 0.55 g/g based on gravimetric analysis after termination of the tests (see Table 1). The Wheeler-Jonas dynamic adsorption capacity (W_e_) was calculated to 0.47 g GB/g. The literature contains only limited data on the GB adsorption capacity of AC, but Amitay-Rosen et al.^38^ reported preliminary dynamic, W_e_ and saturated, W_s_ adsorption capacities of 0.31 and 0.34 g GB/g for impregnated carbon (ASC). These values are similar to those obtained here for NU-1000 but only around half of those determined for the nonimpregnated AC used in this work. Impregnation entails adding salts of metals such as copper to AC to increase protection against toxic gases, which reduces its BET-surface area and total pore volume when compared to nonimpregnated AC.^39^

The GB adsorption capacity of AC was almost twice that of the pelletized NU-1000, for which the GB adsorption capacity was determined to be 0.36 g/g based on the 50% point of the breakthrough curve and 0.28 g/g by gravimetric analysis after test termination. Its corresponding W_e_ was 0.24 g GB/g. Son et al. reported a dynamic adsorption loading of 7.0 mmol GB/g for NU-1000 powder, corresponding to an uptake of 0.98 g/g under dry conditions.^24^ This value, which was obtained by calculating breakthrough from the load at saturation via the mass balance, is almost twice that obtained in our analysis. The same authors found that NU-1000 had the highest GB loading of ten different Zr-MOFs and investigated correlations between loading, surface area, and pore volume. Although NU-1000 had both a competitive surface area of 2200 m^2^/g and a total pore volume of 1.52 cm^3^/g, other MOF properties such as SBU-connectivity (i.e., the number of open metal sites) and linker type were found to be more important factors governing GB uptake. The BET-surface areas of the powdered and pelletized NU-1000 used in this study were 2190 m^2^/g and 2350 m^2^/g, respectively (Table S2), indicating that the surface area of our material matched that reported in the literature. In our miniaturized setup, pelletization of the powder was deemed necessary to obtain an acceptable pressure drop at flow rates suitable for the small column diameter of 4 mm. Preliminary tests using MOF powder often yielded irreproducible results, clogging and occasionally instant breakthrough (data not shown).

UiO-66 and many other Zr-MOFs have been successfully pelletized at relatively low pressures without compromising the integrity of the framework or causing significant loss of surface area.^40,41^ The NU-1000 material used here was pelletized by applying a pressure of around 127 MPa for 1 min. Although this corresponded to the lowest practical settings for the press used, BET measurements indicated that this caused loss of micropores (around 12 Å) but not mesopores (around 27 Å) when compared to the powder. It is therefore possible that the material was adversely affected by compression, which may explain why its measured capacity was lower than that reported by Son et al.^24^ Another possible explanation is that synthesis of NU-1000 (**csq** topology) following the protocol of Wang et al.,^35^ also yield a phase of the polymorph NU-901 (**scu**), since the same building blocks are used.^42,43^ According to our characterization data (Figures S1 and S2, Table S2) the relative occurrence of NU-901 in NU-1000 is difficult to determine. Nevertheless, the reported adsorption capacity for GB on NU-901 powder was also found to be high during dry conditions by Son et al. (0.84 g/g).^24^

At a flow rate of 8 mL/min, the miniaturized system achieved a linear velocity of 1.1 cm/s and a gas residence time of 0.19 s. Similar calculations for a typical gas filter canister with a diameter of 10 cm and a 20 mm bed height and with a flow rate of 30 l/min give a velocity around 6.4 cm/s and a gas residence time of 0.31 s. The particle diameter in the miniaturized system was 0.12–0.25 mm as compared to around 2 mm in a typical respiratory filter, making the scale-down (by a factor of 8–16) reasonable in terms of column diameter. Other mesh ranges were not tested experimentally but a narrower range should be advantageous. At the very least, adsorption was not disproportionately favored by an extended residence time in the miniaturized system.

### Breakthrough Experiments under Humid Conditions for Activated Carbon and NU-1000

**Figure 3:**
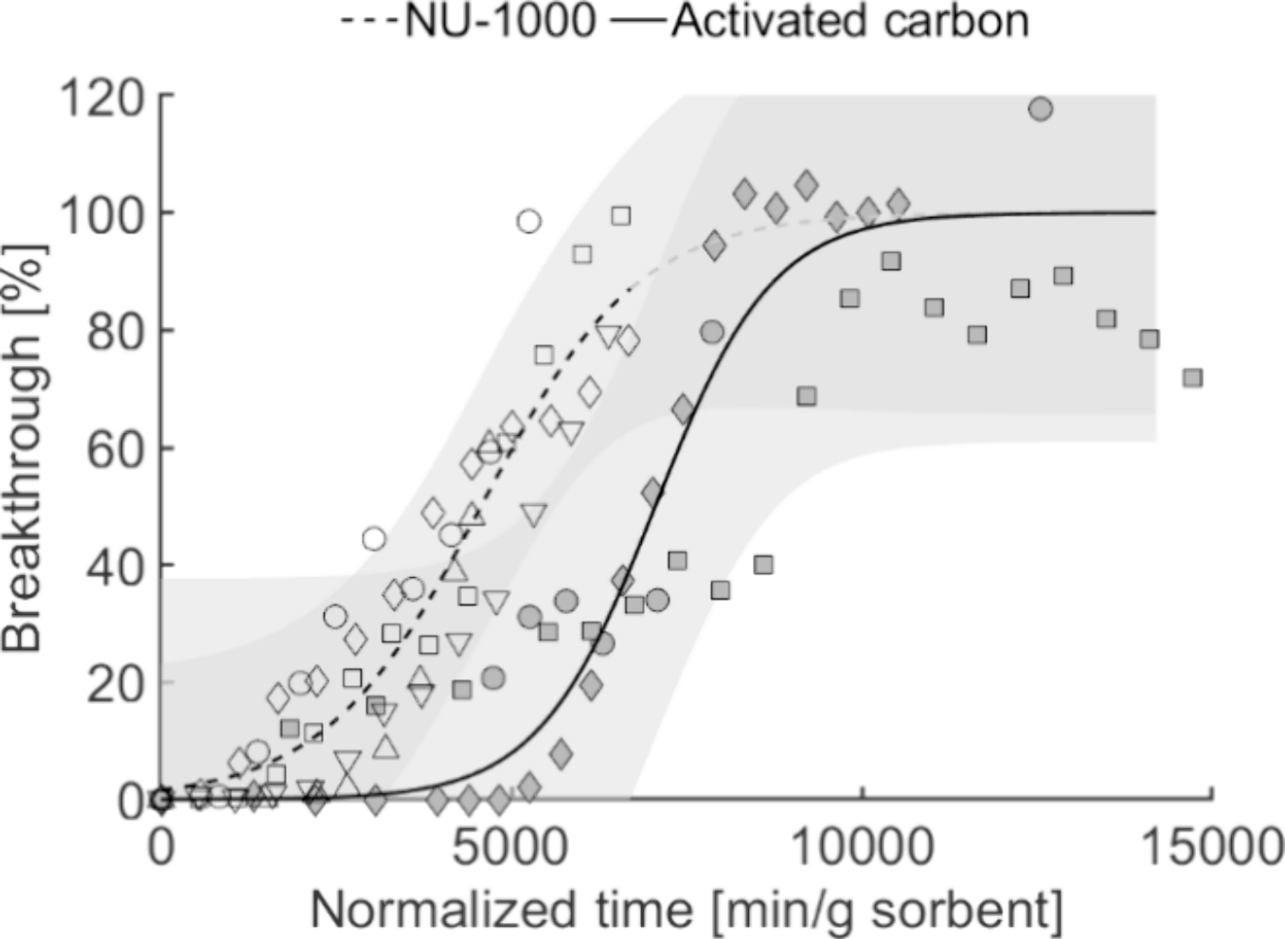
GB breakthrough curves on activated carbon (3 data series, filled symbols) and NU-1000 (5 data series, unfilled symbols) under humid conditions. The gray regions correspond to the 95% confidence intervals for each adsorbent.

**Figure 4:**
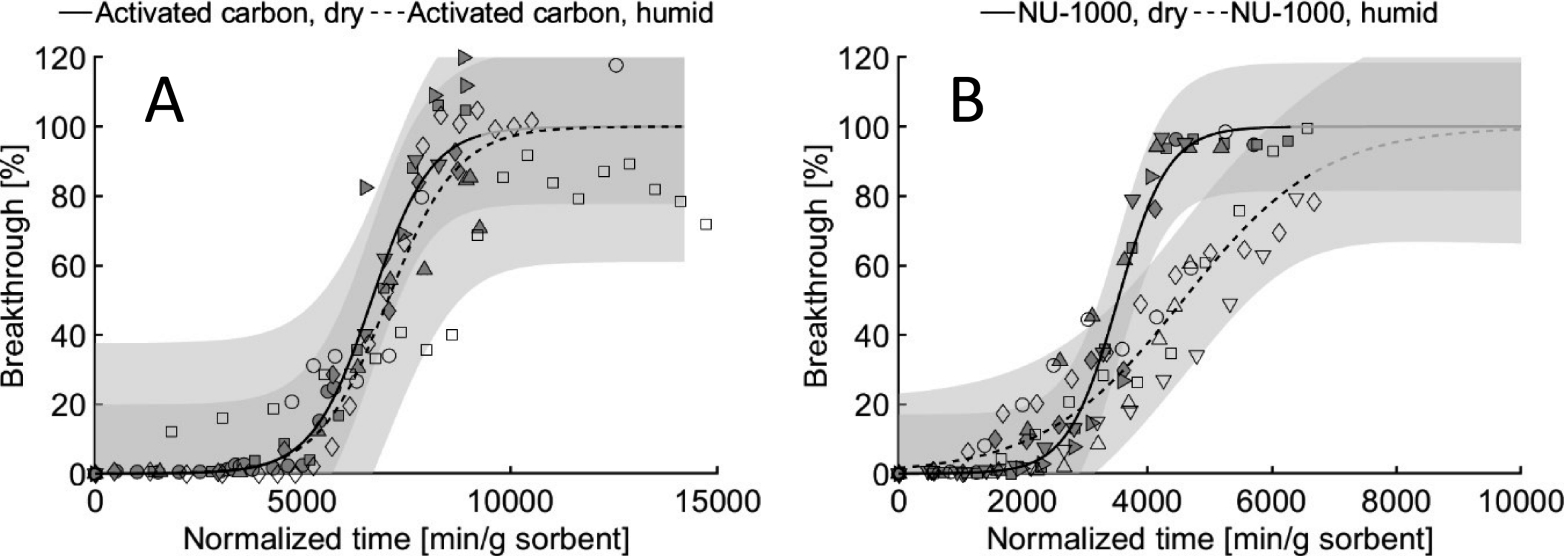
GB breakthrough curves on (A) activated carbon under dry (6 data series, filled symbols) and humid (3 data series, unfilled symbols) conditions and (B) NU-1000 under dry (6 data series, filled symbols) and humid (5 data series, unfilled symbols) conditions. The gray regions correspond to the 95% confidence intervals for each adsorbent. The breakthrough curves for dry and humid conditions are those presented in Figures 2 and 3, respectively.

After obtaining reproducible breakthrough curves for AC and NU-1000 with the miniaturized system, additional tests were conducted under humid conditions. The values of the sorbent mass, pressure drop, water load, GB load at 50% breakthrough calculated from breakthrough curves (Figures 3–4), and gravimetrically determined load after test termination for these experiments are presented in Table 2. The gravimetric GB load was determined after subtracting the calculated average amount of residual water in each tube. As before, the GB adsorption capacity of AC exceeded that of NU-1000 (see Figure 3).

**Table 2:** Dynamic GB Adsorption Capacities from Tests with Activated Carbon (AC) and NU-1000 under Humid Conditions

Sorbent	Mass (mg)	Pressure drop at 8 mL/min (Pa)	Water load^a^ (g/g sorbent)	GB load at 50% breakthrough^b^ (g/g)	GB load at terminated test^c^ (g/g)	Fitted function
AC (*n* = 3)	19.43 ± 1.25	93 ± 30	0.78 ± 0.07	0.72 ± 0.10	0.81 ± 0.07	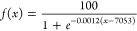
NU-1000 (*n* = 5)	18.70 ± 0.95	75 ± 25	0.67 ± 0.15	0.46 ± 0.10	0.38 ± 0.06^d^	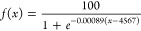

a Water load after prehumidification.

b GB load calculated at 50% breakthrough.

c Gravimetrically determined GB load after subtracting the estimated water content.

d Terminated before saturation.

The adsorption capacities of AC under dry and humid conditions did not differ significantly (Figure 4 A). It is known that a high relative humidity limits the uptake of physiosorbed OP-agents due to competition for available adsorption sites and inhibition of adsorption kinetics.^32,38^ However, in this work the breakthrough curves for AC under dry and humid conditions had similar steepness (k_AC_dry_ = 0.0014 k_AC_humid_ = 0.0012). Amitay-Rosen et al.^38^ similarly found that the breakthrough times for the simulant 2-methoxyethanol on AC were almost identical at 0% RH and 30% RH but decreased from 87 to 65 min at 60% RH. In the same study, the breakthrough time for GB was approximately three times shorter at 85% RH than at 0% RH but no tests at intermediate humidities were performed. It is therefore likely that the negative effect of water is more pronounced at higher RH values.

The breakthrough curve for NU-1000 under humid conditions was flatter than that under dry conditions (*k*_NU-1000_dry_ = 0.0025 vs *k*_NU-1000_humid_ = 0.0009). The difference in adsorption capacity was not significant at 50% breakthrough but there was a shift toward greater GB uptake (see Figure 4B). It was recently discovered that increasing the amount of water in the pores of a structurally similar Zr-MOF, NU-1008, increased the transport and diffusivity of the GB simulant dimethyl methyl phosphonate (DMMP).^44^ Moreover, another study indicated that NU-1000 has a balance of hydrophobicity that both favors water uptake and allows water to participate in hydrolytic reactions at Zr-nodes.^33^ This was attributed to its strong node-linker bonds, large pore volume, and semihydrophobic properties that prevent water from occupying all available sites. The water adsorption isotherm for NU-1000 has been determined experimentally^24,25,33^ and shows a low uptake at lower concentrations followed by a steeper uptake at P/P_0_ > 0.6. This is consistent with a type V isotherm under the IUPAC classification. It was further argued that this type V behavior would be more advantageous than strongly hydrophilic (Type I) or hydrophobic (type III) behavior.^45^ In light of our results, the indications that uptake increased with RH is very interesting.

Several challenges made it difficult to maintain stable experimental settings under humid conditions. Adequate prehumidification of the adsorbents was verified by measuring the change in the adsorbent’s weight and by visual observation of water condensing downstream of the tube. Neither of these methods is particularly accurate because the degree of water condensation on the inner surfaces of the tube was difficult to determine. Furthermore, it emerged that prehumidified NU-1000 dried rapidly when exposed to a flow of dry N_2_ corresponding to a typical breakthrough volume of 1 L. This indicated that a supplementary feed of humid air was needed. To determine whether this effectively weakened the drying effect, prehumidified AC- and NU-1000-sample tubes were weighed continuously over 120 min for NU-1000 and 280 min for AC while applying a continuous feed of humid air.

**Figure 5:**
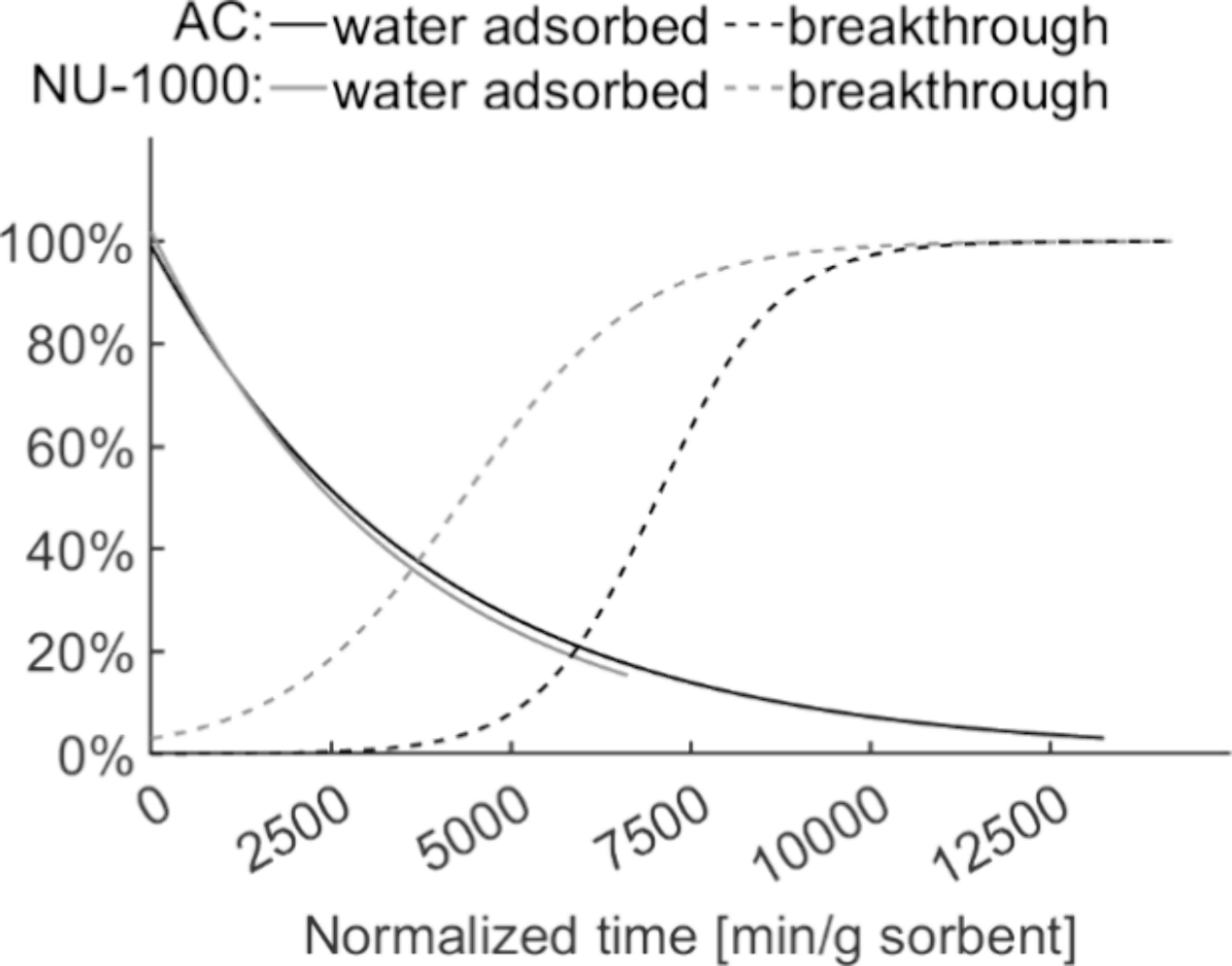
Loss of water content from AC and NU-1000 during breakthrough experiments. Solid lines: water content for AC (black, *n* = 2) and NU-1000 (gray, *n* = 2). Dashed lines: GB breakthrough curves for AC (black) and NU-1000 (gray).

As shown in Figure 5, the water content of both adsorbents declined exponentially at very similar rates under these conditions. Consequently, the adsorbents dried gradually over the course of the experiments even when the analyte flow was supplemented with humid air; the water load of the analyte samples was just 40% of the initial value at the time corresponding to around 50% GB saturation and fell below 20% by the termination of the experiments. For NU-1000, these results are essentially in line with published water isotherms.^24,25,33^ Since none of the materials effectively retained adsorbed water at 55% RH, it can be assumed that humidity levels above 55% RH should be tested to fully evaluate humidity’s effect on their capacity to adsorb and degrade GB.

Another observation made during the breakthrough tests under humid conditions was that the challenge concentration was not reached after saturation: at 55% RH, the breakthrough concentration stabilized at 59% of the challenge concentration when using AC and approached 91% when using NU-1000 (corresponding breakthrough curves are shown in SI, Figure S3). An earlier study indicated that both aerosol formation and condensation may occur when generating gases of water-soluble OP-simulants at concentrations below the saturation pressure at high RH,^32^ and a separate publication highlighted the risk of hydrolysis when generating GB under humid conditions.^24^ This is notable because GB is water-soluble and the experimental conditions applied within the miniaturized analysis system developed in this work were probably close to the thresholds for both condensation and aerosol formation. It would therefore probably be necessary to conduct experiments using lower GB challenge concentrations to fully evaluate the effects of humidity on adsorption; an earlier study used a concentration of 2400 mg/m^3^ at 85% RH,^38^ which is four times lower than the concentration used in our study and could serve as a target concentration for future tests.

### Hydrolysis Measurements

**Figure 6:**
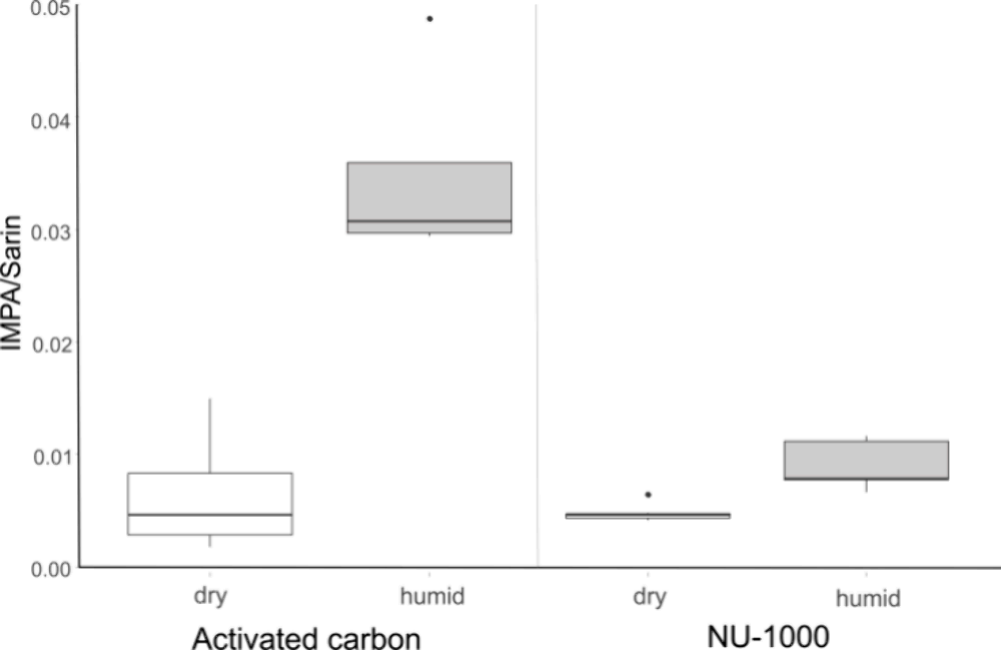
GB hydrolysis based on the IMPA/sarin ratio for activated carbon (AC) and NU-1000 under dry and humid conditions.

Analyses of the IMPA/GB ratio in adsorbent samples collected directly after breakthrough revealed a low degree of hydrolysis (see Figure 6). The highest median degree of hydrolysis into IMPA (3.1%) was achieved with AC under humid conditions. This significantly exceeded the degree of hydrolysis under dry conditions (*p* < 0.05), suggesting a low but measurable rate of water-assisted hydrolysis on the active surfaces of the carbon adsorbent. The ratio of hydrolysis on the NU-1000 samples under humid conditions was also higher than under dry conditions (*p* < 0.05) but in both cases the degree of conversion was below 1%. These results indicate that prerequisites for degradation were not achieved and that the initial amount of water in the adsorbents was insufficient to facilitate significant hydrolysis. Accordingly, an earlier study showed that solid-phase hydrolysis rates are much lower than those in buffer solutions and that raising the water content of NU-1000 from 0 to 400% only slightly increased the hydrolysis rate of the simulant dimethyl 4-nitrophenyl phosphate.^25^ Several recent reviews have highlighted the importance of both water and a suitable base in solid-state hydrolysis;^15,45,46^ even if an OP-agent effectively binds to a hydrated Zr-node and HF is eliminated after nucleophilic attack, a base may still be important for removal of the hydrolysis product. Further, if the hydrolysis product has a high binding energy, the hydrolytic reaction may become stoichiometric rather than catalytic.^47^ Base-assisted removal of IMPA using a base that is either integral to the adsorbent or exogenous was not possible in the tested system, and likely contributes to the overall low degree of hydrolysis.

The analytical method used in this work achieved acceptable performance in terms of reproducibility. No additional experiments were performed to quantitatively evaluate extraction efficiency, but the amount of GB determined by GC/MS was divided by the GB load at the termination of each breakthrough test to estimate the extraction efficiency. This metric gave an estimated extraction efficiency of 126 ± 32%. The analytical method was primarily designed to accurately determine the IMPA/GB ratio, so the IS was added after the extraction step. To more accurately compensate for losses during extraction, it would be desirable to add an appropriate IS before extraction. Nevertheless, the high recovery rate of GB in the samples strengthens the conclusion that the extent of hydrolysis into IMPA was very limited. Since extraction efficiencies of alkylphosphonic acids, and especially methyl phosphonic acid(s), from solid samples are reported to vary,^48,49^ dedicated tests designed to assess extraction efficiency from Zr-MOFs could be useful.

To summarize, the main purpose of this study was to develop a screening method for assessing the ability of Zr-MOFs to adsorb and hydrolyze the highly toxic OP-agent GB in the gas phase. The developed method was inspired by the conditions used in dynamic tests that are commonly used to assess respiratory filters with less toxic simulants or toxic industrial chemicals (TIC). Because the accessibility of both MOFs and GB is limited, the aim was to develop a scaled-down method that would be simple, inexpensive, and safe. Before starting tests with GB, the adsorption capacity and breakthrough times of cyclohexane on two AC size fractions were determined using both the miniaturized test system presented here and a more conventional larger system. The adsorption capacities in both cases were similar (0.33 g/g and 0.25 g/g, respectively).^50^ Small volatile hydrocarbons such as cyclohexane are frequently used to investigate adsorption properties related to physiosorption,^51^ while organic phosphonates such as DMMP are used to mimic GB in adsorption studies because of their similar chemical properties, kinetic diameters, and content of polar functional groups.^52^ However, GB is less stable than many OP-simulants used to assess adsorption behavior because its strongly electronegative fluorine center becomes an excellent leaving group.^53^ This makes routine testing with GB difficult and may be one reason why there are few published experimental studies on the adsorptive uptake of GB.^38^ This work showed that GB adsorption and degradation can be reproducibly evaluated in a miniaturized test system under both dry and moderately humid conditions. The rate of GB hydrolysis was significantly higher under humid conditions for both tested adsorbents, and humidity also interestingly increased the uptake of GB by NU-1000. However, in future it would be interesting to determine whether the degradation of GB on NU-1000 could be improved by increasing the humidity or adding a base. Another important challenge will be to find methods for monitoring the appearance of the major leaving groups from GB during gas-phase hydrolysis in a dynamic set up. However, it will probably be difficult to continuously monitor the formation of minute amounts of hydrogen fluoride, HF_(g)_ and the chemically stable alkylphosphonic acid IMPA_(l)_ due to the former’s high reactivity and the latter’s low volatility.^53^

## Conclusions

A miniaturized method for assessing the adsorption and gas-phase hydrolysis of GB on Zr-MOFs has been developed. Despite using small amounts of both adsorbent material and toxic agent, the method provided reproducible data under both dry and humid conditions. Moreover, the linear velocities and gas residence times observed using the miniaturized system were similar to those seen in large scale test systems for respiratory filters. Experiments were performed using the Zr-MOF NU-1000 (after pelletization) and nonimpregnated AC as adsorbents, revealing that AC had a greater adsorption capacity for GB. Difficulties related to generating GB gas were partly overcome by using deactivated liners and tubing. The results obtained indicated that a lower challenge concentration than that employed in this work should be used when performing experiments at higher humidity levels to prevent losses due to condensation or hydrolysis. Another notable finding is that water may enhance the uptake of GB on NU-1000 and that future tests should therefore be performed at >55% RH and/or with an added base. Neither of the tested adsorbents exhibited a great ability to hydrolyze GB under moderately humid conditions on a short time-scale, but hydrolysis under humid conditions was significantly faster than under dry conditions. Future simulation studies may reveal corresponding mechanisms from the improved performance. Overall, the miniaturized method was shown to be suitable for screening pelletized Zr-MOFs to evaluate their adsorption of OP agents under dynamic conditions. Our results also show that nonimpregnated AC is an efficient adsorbent for gaseous GB and still seems to be an effective material for this purpose.

## Supporting Information Available

Additional experimental details, FTIR spectra, SEM images, and PXRD-data (PDF)

## Abbreviations

AC: activated carbon
ATR FTIR: attenuated total reflectance Fourier-transform infrared spectroscopy
Average CV: average coefficient of variation for independent series of determinations
BET: Brunauer–Emmett–Teller
CEM: controlled evaporator mixer
CV: coefficient of variation
CWC: Chemical Weapon Convention
FPD: flame photometric detector
GB: sarin
HF: hydrogen fluoride
i.d.: inner diameter
IMPA: isopropyl methyl phosphonic acid
IS: internal standard
*k*: curve steepness in logistic function
OP: organophosphorous
OPCW: Organisation for the Prohibition of Chemical Weapons
NU-1000: Zr_6_(_μ3_–OH)_8_(OH)_8_(TBAPy)_2_
RH: relative humidity
PXRD: powder X-ray diffraction
SEM: scanning electron microscopy
TD-GC/FID: thermal desorption gas chromatography flame ionization detection
W_e_: Wheeler-Jonas dynamic adsorption capacity
Zr-MOF: zirconium metal organic framework
