# Supplementary material for: A Miniaturized Method for Evaluating the Dynamic Gas-Phase Adsorption and Degradation of Sarin on Porous Adsorbents at Different Humidity Levels
Source: ACS Omega. 2024 Jun 21;9(26):28412–21. doi: 10.1021/acsomega.4c02306 (PMC11223133; doi:10.1021/acsomega.4c02306)
Supplement: Supplementary file 1 — ao4c02306_si_001.pdf [file ao4c02306_si_001.pdf]

Supporting information

A miniaturized method for evaluating the dynamic gas-phase adsorption and degradation of sarin on porous adsorbents at different humidity levels

*Lillemor Örebrand, Linnea Ahlinder, Marianne Thunéll, Robin Afshin Sander, Andreas Larsson, Andreas Fredman, Håkan Wingfors**

Author Address: Swedish Defence Research Agency, CBRN Defence and Security, Cementvägen 20, 901 82 Umeå, Email: hakan.wingfors@foi.se

List of contents

Additional experimental details and characterization data

- S 1.1 Chemicals
- S 1.2 Material preparation
- S 1.3 Instrumentation
- S 1.4 Humidification
- S 1.5 Chemical analysis
  - Table S1 , MS/MS settings for determination of IMPA
- S 1.6. Material characterisation
- S 1.7 Characterization of NU-1000 and pelletized NU-1000
  - Figure S1. Scanning electron microscopy (SEM)
  - Figure S2. ATR-FTIR spectra on NU-1000 and PXRD data.
- S 1.8 Breakthrough curve without normalization
  - Figure S3
- S 1.9 Wheeler-Jonas
- References

S1.1 Chemicals

In addition to the chemical warfare agent sarin (>98 % purity), a wide range of chemicals were used in this work. Nitrogen was used as a carrier gas (Linde Nitrogen Chemical 4.6, ≥ 99.996 %) and air was filtered through activated carbon before adding deionized water to establish the desired humidity.

Hydrolysis measurements were performed using dry acetonitrile (Merck, Germany), 2,4-dichloroaniline (99 %, Merck, Germany) as an internal standard, and mobile phases containing ammonium acetate (Merck, Germany) and acetonitrile.

S1.2 Material preparation

H4TBAPy and NU-1000 was synthesised in-house following the protocol of Wang et. Al (2016), giving a yellow powder. In short, ZrOCl2 and benzoic acid in DMF was sonicated until fully dissolved before heated in an oven at 100 °C for 1h. H4TBAPy in DMF was treated in the same manner in a separate bottle, before mixed together with the zirconium solution and heated in an oven at 120 °C for 16 h. The precipitated NU-1000 was isolated through centrifugation with repeated washings of DMF, treated with HCl, and finally stored under fresh DMF until used. Before usage, NU-1000 was repeatedly washed with acetone and then activated in an oven at 120 °C for 12 h. The product's identity was verified by scanning electron microscopy (SEM) using a Phenom ProX table-top instrument (ThermoFisher, Germany), ATR-FTIR (Bruker Alpha FTIR), XRD (Phaser D2, Bruker, Germany), and Brunauer-Emmett-Teller (BET) surface area analysis **was** performed at Ångström Laboratories, Uppsala University (Table S2, Figure S2).

The NU-1000 powder was manually pelletized using Perkin-Elmer Hydraulische Presse at the lowest possible pressure (~127 MPa for approximately 1 minute) to obtain durable pellets, followed by careful grinding in a mortar with a pestle and sieving with Newark standard test sieves (sieve no. 60, 250 µm opening, and sieve no. 120, 125 µm opening) to obtain the 125-250 µm sieve fraction. Unimpregnated AddSorb GA activated carbon (AC) supplied by Jacobi Carbons, France, was also grinded and sieved to the same size fraction to serve as a reference in the breakthrough experiments.

Steel tubes, 4 mm i.d. were prepared with a supporting metal mesh and a glass microfibre filter disc (punched from Whatman GF/C 25 mm filters) and loaded with 19.7 ± 1.7 mg AC or NU-1000 adsorbent. The pressure drop over the packed column was measured (Special instruments "DIGMA® UNI 3") at air flows of 5, 8, 10 and 20 ml/min to ensure consistent adsorbent packing.

Images of the NU-1000 powder and the pelletized NU-1000 125-250 µm sieve fraction were collected on the above-mentioned SEM instrument and powder samples were sent to Ångström Laboratories at Uppsala University for BET measurements of parameters including surface area, pore size distribution, and pore volume (Table S1).

S 1.3 Instrumentation

Target analytes were detected using a TD-GC-FID system consisting of a TD100-xr thermal desorber (Markes international), a Trace 1600 gas chromatograph with a flame ionization detector (ThermoFisher Scientific) fitted with a DB5 column (30m, 0.53 mm i.d., 0.25 µm film thickness). All samples were analysed using 2-3 stage desorb settings on the Markes TD100-xr: tube desorption 4 min, 250 °C, 15 ml/min. Trap settings 1 min purge, 20 ml/min purge flow, trap low temp 20 °C, maximum heating rate up to 280 °C, total trap desorb time 4 min, trap split flow 30 ml/min. The GC was operated in constant pressure mode (95,8 kPa) and the oven temperature was ramped from 70 °C (hold 1 min) to 300 °C (hold 1 min) at a heating rate of 30 °C/min.

S 1.4 Humid conditions and pre-humidification

A humidifying system was built using mass flow controllers from Bronkhorst, The Netherlands, and connected to an air flow system. Deionized water from a pressurized container was added (50 g/h, Liqui-flow) to a CEM (Controlled Evaporator Mixer) and mixed with dry air (10 L/min, EL-flow). The resulting humid air flow was introduced via a second air flow system, diluting it to a total of 40 L/min. Only 4 ml/min of this humid diluted flow was used in the breakthrough tests.

The same CEM- and air flow systems were used during pre-humidification but in this case 90 g/h deionized water was added to the system. Pre-humidification was conducted by connecting the packed sample tubes perpendicularly to the humid air flow. The humid air (50-60 % RH at 40 °C, ~30 g water/m^3 air) was drawn through the sample tube at 50 ml/min for 20 minutes using a pocket pump (SKC, Pocket Pump Touch).

S 1.5 Chemical analysis

Trap samples (Tenax TA, 60/80 from Markes international, United Kingdom) were used to verify challenge and breakthrough concentrations. When collecting trap samples to measure challenge concentrations, a 39 mm long 4 mm i.d. steel tube was placed between the T-piece and the trap sample to ensure that the distance between the mixing point at the tip of transfer line and the Tenax granulates in the trap sample tube was similar to the distance between the mixing point and the adsorbent in the other tests. During breakthrough tests, trap samples were collected by connecting the trap sample directly to the adsorbent tube using a 1/4” union from Swagelok. The sampling time was 15 seconds and the flow from the sample tube was the only flow through the Tenax tube, meaning that no additional pump was used. All trap samples collected under humid conditions were flushed with ambient air for 2 min at 50 ml/min to remove excess water and reduce the risk of GB hydrolysis between sampling and analysis.

S 1.6 Hydrolysis measurements

After transferring the saturated adsorbent materials to glass vials and adding 2 mL of dry acetonitrile (ACN) the material was extracted by ultrasonication for 20 minutes. After sedimentation, 10 µL of the clear phase was diluted with ACN to a final volume of 1000 µL. The samples were then stored in a freezer (-20 °C) until analysis. Two 10 µL aliquots were withdrawn, one for GB determination and one for isopropyl methylphosphonic acid (IMPA) determination. Both were spiked with 945 ng of the internal standard (IS) 2,4-dichloroaniline before analysis. The GB/IS quota was determined by GC/MS and the IMPA/IS quota by LC/MS.

The GC/MS instrument was an Agilent 7890B/5977MSD operating in EI mode (70 eV) with a DB5 MS column (30 m, 0.25 mm i.d., 0.25 µm film thickness) for separation after injection of a 1 µl sample in splitless mode. The GC was ramped from 60 °C to 280 °C at 15 °C min^{-1} and held for 5 minutes. The m/z ions 99 and 125 were used for quantification and verification of GB (t_R=3.32 min) and m/z ions 161 and 163 were used for IS, 2,4-dichloroaniline (t_R=10.07 min).

LC-MS/MS analyses were performed with an Acquity UPLC I Class Plus instrument coupled to a Xevo TQ MS triple quadrupole mass spectrometer (Waters; Milford, MA, USA). The MS was equipped with an electrospray ionisation source (ESI) and operated in both positive and negative ion mode. The capillary voltage was set to 3 kV in positive ESI and 2.5 kV in negative ESI, and the collision gas was argon. IMPA and DCA were analysed in multiple reaction monitoring (MRM) mode using the settings given in Table S1 below.

The analytes were separated on an Acquity UPLC BEH HILIC, 2.1x100 mm (1.7 µm) column (Waters). Mobile phase A was 20 mM ammonium acetate in water and mobile phase B was ACN. Chromatographic separation was achieved using a gradient of 12 % A for 2 min, followed by a linear gradient to 25 % A over 3 min, rising to 99% A over 1 min and holding at 99 % A for 1 min before returning to 12 % A over 0.1 min and holding for 3 min. The total chromatographic run time was 10

min. The flowrate was 0.3 ml min^{-1}, the column temperature was 40 °C, and the injection volume was 5 µl.

Table S1. MS/MS settings for determination of isopropylmethylphosphonic acid (IMPA) and 2,4-dichloroaniline (DCA).

	**Precursor ion [m/z]**	**Product ion [m/z]**	**Dwell time [s]**	**Cone voltage [V]**	**Collision Energy [eV]**	ESI mode
IMPA (quantifier)	137	95	0.070	25	15	-
IMPA (qualifier)	137	79	0.070	25	30	-
DCA	162	126	0.070	15	20	+
DCA	162	99	0.070	15	25	+

S 1.7 Characterization of NU-1000 and pelletized NU-1000

Scanning electron microscopy (SEM) images of NU-1000 powder and the 125-250 µm NU-1000 sieve fraction are shown in Figure S1. The NU-1000 needles were approximately 6.3 ± 2.6 µm in length (n=15) and the pellet were in the sieve fraction 125-250 µm.

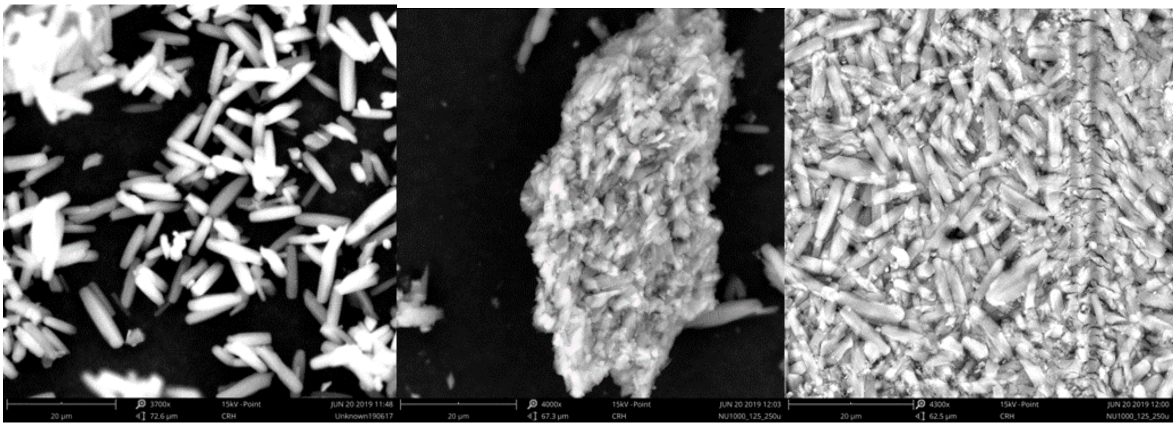

Figure S1. Scanning electron microscopy (SEM) images of NU-1000 powder (left) and two images of particles in the sieve fraction 125-250 µm for NU-1000 (middle and right).

Table S2. Measured BET surface area, pore size and pore volume for NU-1000 powder and the 125-250 µm sieve fraction.

	BET Surface Area (m^2/g)	Pore Size (Å)	Pore Volume (cm^3/g)	Particle size µm
NU-1000 powder	2190	11.8 and 27.6	1.09	6.35±2.64
NU-1000 pellet	2350	27.3	1.97	125-250[a]

[a] sieve fraction

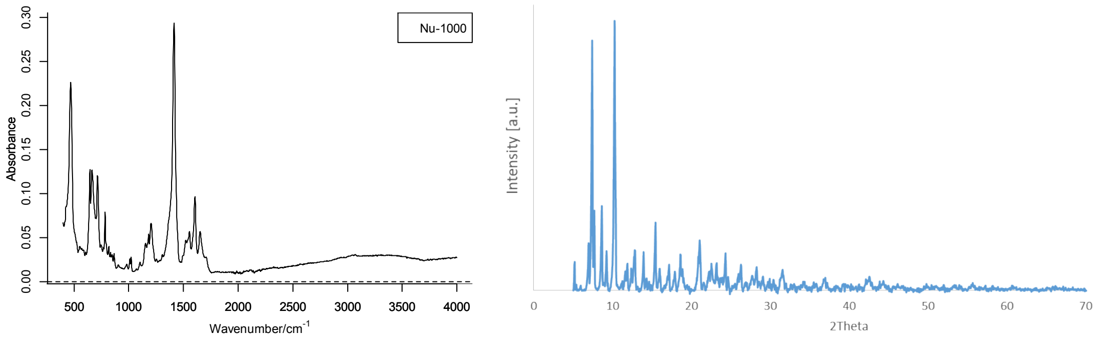

Figure S2. ATR-FTIR spectra and PXRD data for NU-1000.

S 1.8 GB breakthrough curves without normalization

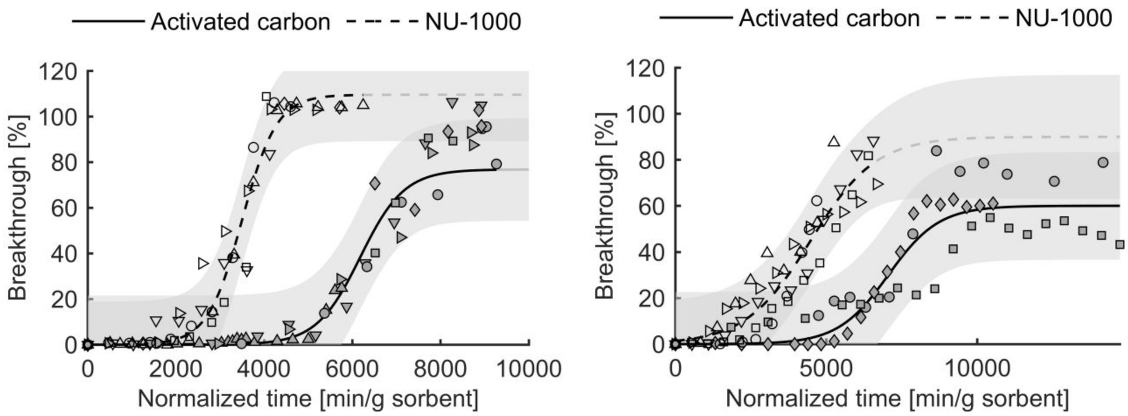

Figure S3. GB breakthrough curves without normalization to 100 %. Left image; activated carbon (6 data series, filled symbols) and NU-1000 (6 data series, unfilled symbols) under dry conditions. Right image; activated carbon (3 data series, filled symbols) and NU-1000 (5 data series, unfilled symbols) under humid conditions. The grey regions correspond to the 95 % confidence intervals for each adsorbent.

S 1.9 Wheeler-Jonas

The Wheeler-Jonas equation allows estimation of breakthrough time of activated carbon through measurable and available parameters through:

$$t_b = \frac{M \times W_e}{Q \times c_{in}} - \frac{W_e \times \rho_b}{k_v \times c_{in}} \times ln\left(\frac{c_{in} - c_{out}}{c_{out}}\right)$$

where t_b is the breakthrough time to reach c_{out} (minutes), M the weight of the adsorbent (g), W_e is the dynamic adsorption capacity (g/g adsorbent). Q is the volumetric flow rate (ml/min), and c_{in} the inlet concentation (g/ml), ρ_b the bulk density (g/ml) and k_v the adsorption rate coefficient (min^{-1}).

By plotting t_b against ln[(c_{in}- c_{out})/ c_{out}], W_e can be calculated from the intercept and k_v from the slope(Lodewyckx et al. 2004). Data and results for Wheeler-Jonas calculations for breakthrough curves of NU-1000 and AC are found in table S3. Problems with reaching c_{in} for tests with AC during humid conditions resulted in large variation using Wheeler-Jonas-calculations.

Table S3. Wheeler-Jonas, data and calculated values for W_e and k_v.

**Material**	**n**	***M* (g)**	**ρ_b (g/ml)**	**W_e (g/g)**	**k_v (min^{-1})**	**r^2**
AC(dry)	4	0.021	0.42	0.47 ± 0.06	1387 ± 83	0.87
AC (humid)[b]	*2*	*0.020*	*0.42*	*0.80 ± 0.49*	*775 ± 197*	*0.86*
NU1000(dry)	5	0.020	0.54[a]	0.24 ± 0.03	1975 ± 262	0.91
NU1000 (humid)	5	0.020	0.54[a]	0.31 ± 0.08	1560 ± 458	0.89

[a] calculated from density data in Islamoglu et (2018) with a product containing 25 % phase of NU-901. [b]poor fit of data due to problems to reach c_{in} during breakthrough experiments.

References

Islamoglu, T.; Otake, K.; Li, P.; Buru, C. T.; Peters, A. W.; Akpinar, I.; Garibay, S. J.; Farha, O. K. Revisiting the structural homogeneity of NU-1000, a Zr-based metal-organic framework. Crystengcomm 2018, 20 (39), 5913-5918. DOI: 10.1039/c8ce00455b.

Wang, T. C.; Vermeulen, N. A.; Kim, I. S.; Martinson, A. B. F.; Stoddart, J. F.; Hupp, J. T.; Farha, O. K. Scalable synthesis and post-modification of a mesoporous metal-organic framework called NU-1000. Nature Protocols 2016, 11 (1). DOI: 10.1038/nprot.2016.001.

Lodewyckx, P.; Wood, G. O.; Ryu, S. K. The Wheeler-Jonas equation: a versatile tool for the prediction of carbon bed breakthrough times. Carbon 2004, 42 (7), 1351-1355. DOI: 10.1016/j.carbon.2004.01.016.
